# Ultrasound-assisted extraction of *Hibiscus rosa-sinensis* flower extracts to be utilized as a sustainable ingredient for the development of functional tea

**DOI:** 10.1016/j.ultsonch.2025.107628

**Published:** 2025-10-17

**Authors:** Hassan Raza, Muhammad Tauseef Sultan, Samran Khalid, Muhammad Usman Khalid, Kashmala Chaudhary, Shehnshah Zafar, Ahmad Mujtaba Noman, Ahmad Raza, Helen Onyeaka

**Affiliations:** a Faculty of Food Science and Nutrition, Bahauddin Zakariya University Multan, Pakistan; b Food Science Program, Division of Food, Nutrition and Exercise Sciences, University of Missouri, Columbia, MO 65211, United States of America; c National Institute of Food Science and Technology, University of Agriculture, Faisalabad 38000, Pakistan; d School of Chemical Engineering, University of Birmingham, Birmingham, United Kingdom

**Keywords:** *Hibiscus rosa-sinensis*, Functional tea, Ultrasound assisted extraction, Antioxidants, Sustainable extracts

## Abstract

*Hibiscus rosa-sinensis* flower is gaining attention for their rich nutritional and functional profile, positioning them as promising ingredients for health-oriented functional foods and beverages. However, limited studies have explored their direct application in functional foods and beverages. This study addresses that gap by assessing the nutritional composition, antioxidant potential, and incorporation of *H. rosa-sinensis* flower extract as a sustainable ingredient in clean-label functional tea formulations. Ultrasound-assisted extraction (20  kHz, 40 °C, 30  min; 1:20 solvent ratio) using various solvents was employed to obtain flower extracts. Functional teas formulations were developed by replacing green tea with flower extract at 25 %, 50 %, 75 %, and 100 % levels. Nutritional analysis of *H. rosa-sinensis* flower revealed high carbohydrate content (61.50 ± 1.18 %) and ash (3.71 ± 0.29 %), with calcium (1218 ± 26  mg/100  g), and potassium (1109 ± 40  mg/100  g) as dominant minerals. The *H. rosa-sinensis* flower extract exhibited strong antioxidant properties with total phenolic content (TPC) of 781.77 ± 4.66  mg GAE/100  g, DPPH inhibition activity of 84.72 ± 2.48 %, ABTS activity of 90.01 ± 2.32 %, and FRAP activity of 1654.4 ± 15.62  µg Fe/100  g. In tea formulations, the highest TPC and FRAP activity were in the control that was 100% green tea (429.98 ± 5.16  mg GAE/100  g, 1759.0 ± 4.48  µg Fe/100 g), while 75 % *H. rosa-sinensis* extract containing functional tea showed the highest DPPH inhibition activity of 70.05 ± 1.48 %, and ABTS activity of 71.79 ± 1.54 %. The sensory analysis indicated that the 50 % *H. rosa-sinensis* extract containing functional tea had the highest acceptability and optimal color attributes. FTIR analysis confirmed the presence of key functional groups (O–H, C=C), indicating abundant polyphenols and flavonoids. Overall, *H. rosa-sinensis flower* demonstrated excellent potential as sustainable ingredient for functional food and beverages development, offering improved antioxidant benefits and appealing sensory properties.

## Graphical abstract

**Figure:**
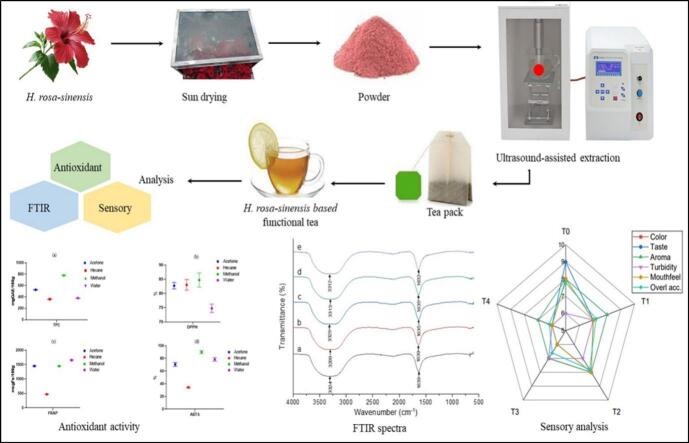

## 1 Introduction

The modern food industry faces rising consumer demand for health, nutrition, and sustainability, prompting a shift toward clean-label, and functional foods and beverages made with natural, sustainable, and recognizable ingredients, free from artificial additives. Functional foods and beverages are enriched with bioactive components like prebiotics, vitamins, minerals, antioxidants, herbal extracts, amino acids, and plant-based compounds, which offer functional benefits beyond basic nutrition by helping prevent or manage diseases [1]. The functional foods and beverages market is among the fastest-growing segments of the food industry, largely due to increasing health concerns associated with the widespread consumption of convenient foods, self-medication practices, and sedentary lifestyles [2]. The production of functional beverages has been on the rise by beverage industries as the market of functional beverages in 2024 was 210 billion USD and it is expected to grow to 339.6 billion USD by 2030 [3]. Different functional beverage categories may include drinks for weight management, immunity, cognitive, gastrointestinal and cardiovascular health [4].

Functional tea among the functional beverages, is labelled as caffeine-free herbs blend or mixture which imparts multiple health benefits upon consumption. Different teas consumption which are infused with flowers, leaves, fruits and seeds of various herbs, plants, and vegetables are widely practiced to transmit good health effects and alleviation of oxidative stress along with their hydric status because of their phytochemical composition [5]. *Hibiscus rosa-sinensis*, an evergreen herbaceous medicinal plant, belongs to the Malvaceae family and Hibiscus genus that has around 275 species in tropical and subtropical regions of the world [6]. *H. rosa-sinensis* flowers exhibit strong antioxidant activity as a dietary source, with polyphenol content comparable to that of green tea and grapefruit juice [7]. Moreover, they have also shown different health benefits, including anticancer [8], anti-diabetic [9], anti-inflammatory [10], cardio-protective [11], and anti-microbial [12] among others. *H. rosa-sinensis* despite exhibiting various health benefits have remained underexplored in contrast to *H. sabdariffa* which is studied extensively for its phytochemical composition and functional applications. Some unique compounds (e.g., cyanidin-sophoroside) present in *H. rosa-sinensis* are absent in *H. sabdariffa* [13,14]. These compositional differences may impart distinct nutritional and functional attributes, justifying the selection of *H. rosa-sinensis* for the development of functional tea in the present study. So, these flowers are being used but should be used more extensively in the production of pharmaceuticals, cosmetics, foods, and beverages. Their vibrant red to maroon color, berry-like aroma, mildly acidic taste, and distinctive floral morphology can make them particularly appealing for such applications [15].

Various *H. rosa-sinensis* products such as hot and cold teas, ready-to-drink formulations, infusions, herbal teas, diluted extracts, and syrups have gradually gained popularity across different regions of the world. Over the past few decades, these products have appealed to health-conscious consumers, those seeking novel, vibrant, exotic, or all-natural drinks, as well as ethnic communities familiar with traditional uses of the flower [7]. The recovery of extracts from these flowers is a crucial step toward their application as natural sustainable ingredients in food, cosmetics, and pharmaceuticals. While conventional extraction methods have been extensively employed, they are often associated with limitations such as low selectivity, solvent residues, and high energy consumption. These drawbacks have driven the exploration of sustainable, eco-friendly, and clean-label extraction techniques. Among emerging techniques, ultrasound-assisted extraction (UAE) has garnered considerable interest due to its enhanced extraction efficiency, high mass transfer, increased yield, reduced processing time, lower solvent and energy consumption, minimal waste generation, and lower environmental impact [16]. Specifically, for *H. rosa-sinensis*, UAE preserves heat-sensitive bioactive compounds such as polyphenols and flavonol glycosides, resulting in extracts with preserved bioactivity, which is very important [17,18]. Moreover, UAE is easily scalable because ultrasonic reactors can be adapted from laboratory to industrial scale, allowing large-volume processing with consistent quality. While the initial cost of ultrasonic extraction equipment is higher than conventional methods, UAE offers lower operational costs due to reduced extraction times, decreased solvent usage, and higher bioactive yields [19]. This makes it economically attractive for producing high-value functional teas, where product quality and clean-label processing can justify the investment [20]. Additionally, the sustainable nature of UAE further reduces long-term production and environmental costs. These advantages make UAE particularly suitable for optimizing bioactive recovery and sensory attributes in novel functional beverages [16,21].

This study explores the potential of an underutilized hibiscus specie, *H. rosa-sinensis* for functional tea development. While *H. sabdariffa* has been extensively studied, the unique combination of bioactive compounds, sensory properties, and the use of sustainable extraction technique in this study positions *H. rosa-sinensis* as a promising candidate for clean-label functional beverages. This study optimizied the extraction of bioactive compounds rich extracts from *H. rosa-sinensis* flowers using UAE with different solvents. The research evaluates the proximate composition and mineral content of the flowers, total phenolic content (TPC), and antioxidant activity using various analytical methods of flower extracts obtained through UAE. In the next phase, functional teas were formulated using different ratios of flower extract. These teas formulations were assessed for TPC, antioxidant activity through multiple assays, color attributes, fourier transform infrared spectroscopy (FTIR), and sensory characteristics by an expert panel. To the best of our knowledge, this is the first study to utilize sustainably extracted *H. rosa-sinensis* flower extracts via UAE for the development of a clean-label functional tea.

## 2 Materials and methodology

### 2.1 Raw materials

*H. rosa-sinensis* flowers were handpicked in the morning from the vicinity of Multan, Pakistan. All the flower samples were collected into transparent polypropylene bags. All the flowers were fully blossomed, picked manually from the plant and they were without any disease, blemish or spot. Identification of the plant leaves was performed by Prof. Dr. Zafar Ullah (Botanist, Department of Botany, BZU, Multan, Pakistan) with the voucher number assigned as https://www.wfo.org/taxon/wfo-0000723007. All the flower were collected into transparent polypropylene bags. All the chemicals used in the study for nutritional analysis, antioxidants, and product development were of analytical grade (Merck KGaA, Darmstadt, Germany).

### 2.2 Preparation of flower powder

The collected flowers were gently rinsed with distilled water to remove any extraneous impurities. The samples were then carefully placed in a specially designed solar dryer operating under standardized conditions. The dryer was equipped with a temperature control system (≤45–50 °C), an automated humidity control regulator, and exhaust ventilation to ensure smooth air circulation and uniform solar heat distribution. The relative humidity was maintained between 18–23 % and continuously monitored using a digital thermo-hygrometer (Extech-445703, Nashua, USA). Drying was carried out during peak sunlight hours (10:00 am–4:00 pm) with samples arranged in a single layer on a breathable mesh to facilitate consistent airflow. Loading density for the fresh flowers was set at 4–4.5 kg/m^2^, with drying period of 7 ± 0.5 hr for each batch. Similar conditions of temperature, humidity and drying duration were given to all the prepared batches to ensure uniform moisture content across batches. Drying was performed until samples reached constant moisture content of ∼ 10 % to ensure drying reproducibility without affecting bioactive compounds retention. The dried flowers were subsequently milled into powder and stored in food-grade bags for further use.

### 2.3 Nutritional analysis of *H. rosa-sinensis* flower

#### 2.3.1 Proximate analysis

Proximate analysis of dried *H. rosa-sinensis* flower was conducted using AOAC (2016) standard methods: moisture (925.10), ash (942.05), crude fat (934.01), crude fiber (978.10), and crude protein (984.13).

#### 2.3.2 Mineral analysis

For the mineral analysis, flower was determined for calcium, zinc, iron, potassium, sodium and magnesium by using flame photometer (410, Sherwood Scientific Ltd, Cambridge, UK) and atomic absorption spectrometry (iCE 3000 Series, Thermo Scientific, Waltham, MA, USA) by following procedure of Huang et al. [22]. A 5 g sample of *H. rosa-sinensis* flower powder was placed in a conical flask, and 10 mL of concentrated nitric acid was added. The flask was heated on a mantle at 85 °C until yellow fumes appeared. Subsequently, 6 mL of concentrated perchloric acid was added, and the mixture was further heated to 180 °C with continuous stirring until the evolution of yellow fumes resumed. The resulting solution was filtered, and the final volume was made up to 100 mL using distilled water. The prepared samples were then analyzed using the previously mentioned equipment.

### 2.4 Ultrasound assisted extraction of *H. rosa-sinensis* flower extract

**Fig. 1:**
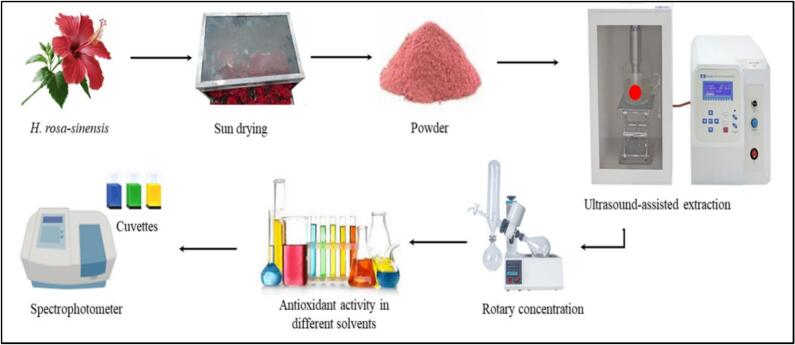
Schematic representation of the preparation of *H. rosa-sinensis* flower, including drying and grinding to obtain powder, followed by ultrasound-assisted extraction to obtain extracts.

UAE was performed using the method of Covarrubias-Cárdenas et al. [23] with slight modifications. Samples were mixed with different solvents (acetone, hexane, methanol and distilled water) to ensure comprehensive and diverse extraction profiling because of different polarities capturing both lipophilic and hydrophilic fractions, at a 1:20 ratio and then placed in a beaker inside the ultrasonic chamber [24]. A probe-type ultrasonicator (Model VCX130, Sonics & Materials Inc., Newtown, USA) operating at a frequency of 20 kHz, with a power density of 98.0 W/cm^2^, was used for this extraction. A high-intensity probe (CV00018) with a 13 mm diameter was immersed in the sample with solvent, operating at a mode of (50 s on and 50 s off) cycle to prevent excessive heat buildup. The entire process was completed in 30 min. Moreover, an ice bath was also employed around the beaker to minimize the loss of solvent and heat-sensitive compounds during the process, which occurred due to the incremental temperature increase during sonication. The temperature was maintained at 40℃ during the whole process. The whole process is shown in Fig. 1. After allowing the stay time, all extracts were filtered using Whatman filter paper. A rotary evaporator (Heidolph Instruments GmbH & Co. KG, Schwabach, Germany) was used to concentrate the extracts.

### 2.5 *H. rosa-sinensis* flower extract analysis

#### 2.5.1 Total phenolic contents (TPC)

The extraction of phytochemicals was performed using a sequential solvent extraction method employing acetone, hexane, methanol, and distilled water as solvents to ensure a comprehensive recovery of both non-polar and polar compounds. The method of Flores-Martínez et al. [25] was used to determine the TPC based on the Folin–Ciocalteu method with minor modification. Briefly, 0.5 mL of the extract was mixed with 1.5 mL of 5 % (w/v) sodium carbonate solution and 1.5 mL of diluted Folin–Ciocalteu reagent (1:10, v/v with distilled water). The mixture was incubated at room temperature for 90 min in the dark. After incubation, the absorbance was measured at 750 nm using a UV–visible spectrophotometer (Infitek, SP-MUV6000, Jinan, Shandong, China). The TPC was expressed as mg GAE per 100 mL of extract.

#### 2.5.2 Antioxidant activity

The antioxidant activity of flower extracts obtained using acetone, hexane, methanol, and distilled water as solvents was determined through DPPH (1,1-diphenyl-2-picrylhydrazyl) radical scavenging assay by employing the method of Agunbiade et al. [26] ABTS [2,2′-azino-bis(3-ethylbenzothiazoline-6-sulphonic acid)] assay was conducted by using the method of Bendaali et al. [27] and Ferric Reducing Antioxidant Power (FRAP) assay by following the method of Benzie et al. [28]. The DPPH assay was conducted by preparing a reaction mixture containing 600 µL of 0.1 mM DPPH solution in methanol, 400 µL of methanolic extract, and 200 µL of distilled water. The mixture was incubated in the dark at room temperature for 30 min. Absorbance was measured at 517 nm using a UV–visible spectrophotometer. The antioxidant activity was expressed as percentage inhibition of DPPH radicals. ABTS•^+^ working solution was prepared by mixing 7 mM ABTS with 2.5 mM potassium persulfate in a 1:1 (v/v) ratio and incubating the mixture in the dark at room temperature for 16 hr. For the assay, 30 µL of the each flower extract was added to 3 mL of the ABTS•^+^ solution, and the absorbance was measured at 734 nm after 6 min. The antioxidant activity was expressed as % inhibition based on a standard Trolox calibration curve. The FRAP reagent was freshly prepared by mixing 25 mL of 300 mM acetate buffer (pH 3.6), 2.5 mL of 10 mM TPTZ (in 40 mM HCl), and 2.5 mL of 20 mM FeCl_3_·6H_2_O. In the assay, 200 µL of the each flower extract was added to 1.8 mL of the FRAP reagent. The mixture was incubated at 37 °C for 10 min. After cooling to room temperature, the absorbance was recorded at 595 nm. The FRAP activity was expressed as μmol Trolox equivalents (TE) per mL of sample.

### 2.6 Development of *H. rosa-sinensis* flower extract based functional tea formulations

**Fig. 2:**
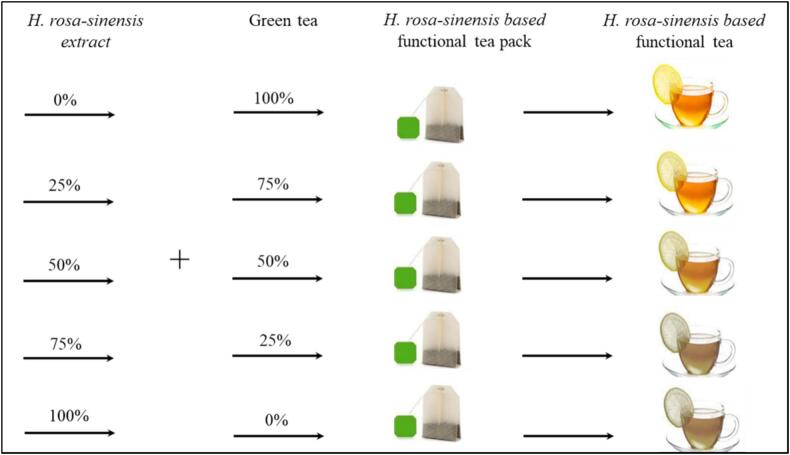
Stepwise illustration of the preparation process for functional tea formulations using *H. rosa-sinensis* flower extract and green tea in varying proportions.

Functional tea formulations were developed using different concentrations of *H. rosa-sinensis* flower extract in combination with green tea, as outlined in Table 1. Each tea bag (approximately 2 g total weight) was prepared by combining the designated ratios of *H. rosa-sinensis* flower extract and green tea. To brew the tea, each tea bag was dipped and stirred in hot water (95–100 °C) for 1 min. The control treatment (T0) consisted of 100 % green tea, while the other treatments involved replacing green tea with 25 %, 50 %, 75 %, and 100 % *H. rosa-sinensis* flower extract, respectively. After brewing, the tea infusion was filtered and allowed to cool to 50 °C before further analysis. The preparation steps for functional tea formulations are illustrated in Fig. 2.

**Table 1:** Treatment plan for *H. rosa-sinensis* extract based functional tea formulations development.

**Treatments**	***H. rosa-sinensis* flower extract %**	**Green Tea (%)**
T_0_	−	100
T_1_	25	75
T_2_	50	50
T_3_	75	25
T_4_	100	−

### 2.7 Analysis of functional tea formulations

#### 2.7.1 Total phenolic contents (TPC)

Flores-Martínez et al. [25] method was used for the determination of the TPC of the functional tea formulations, as outlined in Section 2.5.1.

#### 2.7.2 Antioxidant activity

The antioxidant activity of the tea formulations was determined using three methods, as described in Section 2.5.2, the DPPH assay following the method of Agunbiade et al. [26], the ABTS assay according to Bendaali et al. [27] and the FRAP assay based on the method of Benzie et al. [28].

#### 2.7.3 Colour measurement

The colour analysis of the functional tea formulations was performed by using colour spectrophotometer (Model YS3010, Shenzhen 3NH Technology Co., Ltd., Shenzhen, China). The instrument measured the colour parameters L* (ranging from black to white), a* (green to red), and b* (blue to yellow).

#### 2.7.4 Fourier transform infrared spectroscopy (FTIR)

FTIR analysis of the functional teas formulations for functional groups spectra was carried out by using FTIR spectrometer (Alpha-R, Bruker Optik GmbH, Ettlingen, Germany) with Platinum crystal ATR. Spectra were recorded in absorbance mode over the wavelength range of 400–4000 cm^−1^. A spectral resolution of 8 cm^−1^ was applied, with 32 scans per sample conducted to enhance the signal-to-noise ratio. The resulting spectra were processed using Origin software.

#### 2.7.5 Sensory evaluation

The functional tea formulations were evaluated for sensory attributes using a 9-point hedonic scale, where 1 represented “extremely dislike” and 9 represented “extremely like” as per the methodology of Atlaw et al. [29]. The evaluation was conducted through sensory descriptive analysis by a panel of 20 trained members (aged 25–55 years), selected based on their prior experience in sensory and food evaluation studies and regular participation in taste panels. All panelists underwent a short training session to familiarize them with the evaluation protocol and terminology. Prior to participation, each panelist received a clear explanation of the study’s objectives and signed an informed consent form. The sensory evaluation was conducted in accordance with ethical provisions, and approval was obtained from the Institutional Research Ethics Committee (REC) Review Board. Participation was voluntary, and panelists were informed of their right to withdraw at any time without consequences. Functional tea samples were freshly prepared in transparent cups and presented at room temperature (22 ± 2°C). Each sample was labeled with a random three-digit code to prevent bias. The evaluation environment was designed to be quiet, well-lit, and free from distractions. Panelists assessed each sample for color, aroma, flavor, turbidity, mouthfeel, and overall acceptability. Room-temperature water and plain crackers were provided to cleanse the palate between evaluations. A 2-min interval was maintained between successive samples to minimize sensory fatigue and bias. Sensory scores were expressed as mean ± standard deviation for each attribute.

### 2.8 Statistical analysis

Statistical analysis was performed using Statistix 8.1 software. One-way analysis of variance (ANOVA) was employed to determine significant differences among the mean values of triplicate measurements. Pairwise comparisons were conducted using the Least Significant Difference (LSD) test. All results are reported as means ± standard deviations, and differences were considered statistically significant at *p* ≤ 0.05.

## 3 Results and Discussion

### 3.1 Proximate analysis of *H. rosa-sinensis* flower

The proximate analysis of *H. rosa-sinensis* flower demonstrates a well-defined nutritional profile, indicating their potential for diverse applications. As shown in Table 2, the flowers exhibited a relatively low moisture content of 12.74 ± 0.57 %. The ash content, representing the total mineral composition, was 3.71 ± 0.29 %, while the fat content was measured at 2.10 ± 0.33 %. The crude fiber content was notably higher at 17.42 ± 0.61 %, suggesting potential benefits for digestive health. The protein content was recorded at 2.53 ± 0.19 %. Interestingly, the flowers contained a significantly high carbohydrate content of 61.50 ± 1.18 %, positioning them as a promising source of energy. Similar proximate analysis results were observed in the findings of [30] as this study was conducted with different drying methods involved for the *H. rosa-sinensis* flower including the solar dried method similar to our research, and their study reported protein content of 3.49 %, ash or mineral content of 4.11 % and moisture content of 7.21 % of the flower. Udo et al. [31] evaluated the proximate composition of *H. rosa-sinensis* leaves and reported a significant amount of carbohydrates (31.66 %), lipids (9.60 %), and protein (7.01 %), while having a low content of fiber (3.99 %) and ash (3.07 %). However, based on the literature review, the variation in the proximate composition of *H. rosa-sinensis* is prevalent across different regions. This can be attributed to the differences in environmental conditions, genetic variations, and soil conditions.

**Table 2:** Proximate composition of *H. rosa-sinensis* flower.

**Proximate**	**Values**
Moisture (%)	12.74 ± 0.57
Ash (%)	3.71 ± 0.29
Fat (%)	2.10 ± 0.33
Fiber (%)	17.42 ± 0.61
Protein (%)	2.53 ± 0.19
Carbohydrate (%)	61.50 ± 1.18
Calcium (mg/100 g)	1218 ± 26.32
Zinc (mg/100 g)	17.03 ± 0.07
Iron (mg/100 g)	25.4 ± 0.15
Potassium (mg/100 g)	1109 ± 40.15
Sodium (mg/100 g)	51.54 ± 0.91

Data are expressed as means ± SD.

### 3.2 Mineral analysis of *H. rosa-sinensis* flower

The mineral composition of *H. rosa-sinensis* flower, presented in Table 2, indicates a notably high content of calcium and potassium, measured at 1218 ± 26.32 mg/100  g and 1109 ± 40.15 mg/100  g, respectively. Additionally, the flower contain appreciable amounts of iron (25.4 ± 0.15 mg/100  g) and zinc (17.03 ± 0.07 mg/100  g). The sodium content was determined to be 51.54 ± 0.91 mg/100  g. Our results align with the study of Trivellini et al. [32], who analyzed the distribution of macro- and micronutrients in various parts of *H. rosa-sinensis* flower. They found the highest potassium content (6 mg/organ) in the petals of single flowers at the open flower stage, while iron content was reported at 16 µg/organ in petals. Calcium was most concentrated in the ovary, whereas magnesium showed the highest levels (0.48 mg/organ) in fully open petals. Additionally, the mineral profile of *H. rosa-sinensis* leaves, as studied by Udo et al. [31], revealed the presence of calcium (772.57 ± 0.01  mg/100  g), potassium (181.00 ± 0.50  mg/100  g), phosphorus (42.38 ± 0.01  mg/100  g), sodium (0.33 ± 0.09  mg/100  g), and manganese (2.40 ± 0.03  mg/100  g).

### 3.3 Total phenolic content (TPC) and antioxidant activity of *H. rosa-sinensis* flower extract

**Fig. 3:**
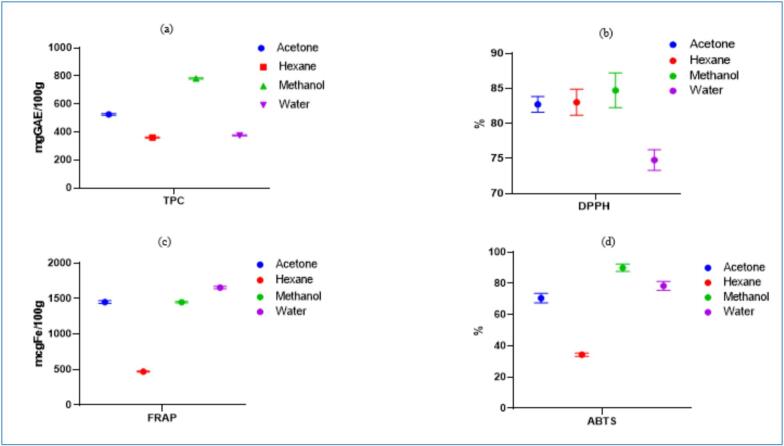
(a) TPC, (b) DPPH, (c) FRAP, (d) ABTS, of *H. rosa-sinensis* flower in different solvents acetone, hexane, methanol and water.

TPC and antioxidant activity of the *H. rosa-sinensis* flower extracted using various solvents are illustrated in Fig. 3. TPC indicates the total concentration of phenolic compounds, which are known for their health-promoting properties, particularly their antioxidant potential. As shown in Fig. 3 **(a)**, the *H. rosa-sinensis* flower extract exhibited the highest TPC in methanol extract, with a value of 781.77 ± 4.66  mg GAE/100  g. Acetone and water extracts showed moderate values of 525.13 ± 5.23 and 375.42 ± 3.62  mg GAE/100  g, respectively. The lowest TPC was observed in the hexane extract, with a value of 360.22 ± 4.25  mg GAE/100  g. The study, conducted by Trivellini et al. [32], also reported a high TPC of 14.4  mg/g on a dry weight basis. Furthermore, Falade et al. [33] found significantly higher TPC values in ethanol and aqueous extracts of *H. rosa-sinensis* flower, reporting 4598.16 and 5436.23  mg GAE/100  g, respectively. The observed differences in TPC among solvents can be attributed to differences in compound solubility, solvent polarity, and solvent-matrix interactions, as well as the enhanced mass transfer provided by ultrasound. Polar and moderately polar solvents, such as hydro-alcoholic mixtures and acetone, preferentially extract polar phenolic compounds, including anthocyanins, whereas non-polar solvents, such as hexane, primarily extract lipophilic compounds and yield minimal phenolics. Similar solvent-dependent extraction patterns have been reported for *H. sabdariffa*, where hydro-alcoholic and acetone extracts exhibited the highest TPC, while hexane extracts showed the lowest [34,35]. These findings are consistent with the polarity and solubility characteristics of the target phytochemicals in *H. rosa-sinensis*. The high polyphenol content of *H. rosa-sinensis* flower suggests its potential application in herbal or functional teas, serving as a valuable dietary source of polyphenols capable of protecting the body against oxidative stress.

The results depicting the values of DPPH (%) inhibition of *H. rosa-sinensis* flower extract in different solvents are presented in Fig. 3 **(b)**. *H. rosa-sinensis* flower exhibited the lowest DPPH inhibition of 74.77 ± 1.47 % in water, while the values in other solvents were comparable: 84.72 ± 2.48 % in methanol, 83.03 ± 1.86 % in hexane, and 82.72 ± 1.13 % in acetone. Our findings align with those of Hamrita et al. [36], who reported that *H. sabdariffa L. calyx* extracts scavenged 86 % of DPPH radicals. Similarly, Falade et al. [33] observed high antioxidant and DPPH activity in methanolic extracts of *H. rosa-sinensis* flowers, with an IC_50_ value of 43.9 mg/mL. Another study performed by Mak et al. [37] also reported DPPH activity for ethanol and aqueous extracts of *H. rosa-sinensis* flower, with ethanol showing 83.08 % inhibition similar to our other solvent results while their aqueous extract demonstrated a higher inhibition of 97.35 %.

Results of FRAP activity of *H. rosa-sinensis* flower extract in different solvents are demonstrated in Fig. 3 **(c)**, which shows significant (*p* > 0.05) differences in values. The highest value of FRAP is elucidated in water, followed by the acetone and methanol solvents with values of (1654.4 ± 15.62, 1450.2 ± 19.25, and 1448.3 ± 10.54 µgFe/100 g), respectively. Hexane extract showed the lowest value (470.6 ± 6.72 µg Fe/100 g). Our results align with the findings of Tong and Lee [38], who measured the antioxidant activity of white and orange *H. rosa-sinensis* flowers and found that the orange flower exhibited the highest FRAP value (125.38 mg FE/g). However, a study conducted by Mak et al. [37] aligns with our findings, reporting FRAP values of 2349.06 and 2883.23 µmoles Fe (II)/100 g for the ethanol and aqueous extracts of *H. rosa-sinensis* flower, respectively.

The ABTS activity of *H. rosa sinensis* flower extract is displayed in Fig. 3 **(d)**, exhibiting variations in values in different solvents. The highest value was found to be in methanol solvent i.e. 90.010 ± 2.32 %, while the lowest was observed in hexane at 34.330 ± 1.02 %. ABTS values for the solvents of acetone and water are almost at par with the values of (70.523 ± 3.03 % and 78.447 ± 2.93 %) respectively. Our results are comparable to those of Maria et al. [7] who reported ABTS scavenging activity of *H. rosa-sinensis* flower petals at 92.25 ± 8.41 % for the petroleum ether extract and 46.12 ± 4.20 % for the butanol extract. Additionally, Sidhu et al. [39] evaluated the antioxidant potential of *H. rosa-sinensis* leaf essential oil, finding an IC50 value of 860 μg/mL for ABTS scavenging activity.

### 3.4 Total phenolic content (TPC) and antioxidant activity of *H. rosa-sinensis* flower extract based functional tea formulations

**Fig. 4:**
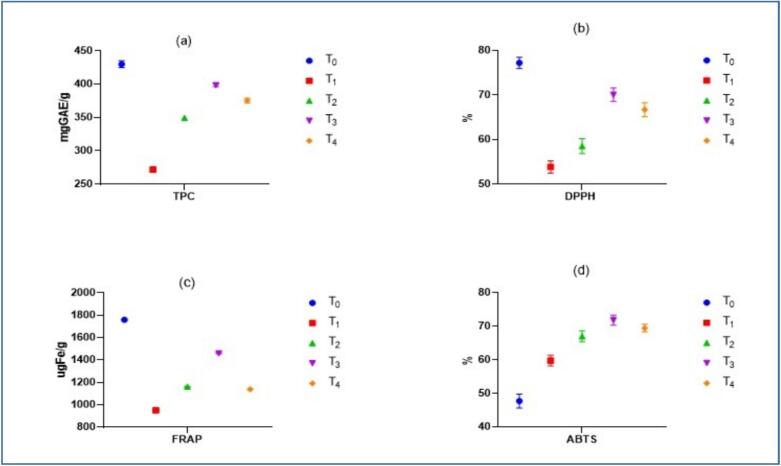
(a) TPC, (b) DPPH, (c) FRAP, (d) ABTS, of *H. rosa-sinensis* flower extract based functional tea formulations. T0 (Control): 100 % green tea, T1: 25 % *H. rosa-sinensis* flower extract + 75 % green tea, T2: 50 % *H. rosa-sinensis* flower extract + 50 % green tea, T3: 75 % *H. rosa-sinensis* flower extract + 25 % green tea, T4: 100 % *H. rosa-sinensis* flower extract.

TPC and antioxidant activity of the *H. rosa-sinensis* flower extract based functional tea with different combinations are presented in Fig. 4. Fig. 4 **(a)** illustrates the TPC of tea formulations prepared using various combinations of *H. rosa-sinensis* flower extract and green tea. The negative control (T0), consisting of 100 % green tea, exhibited the highest TPC value of 429.98 ± 5.16  mg GAE/100  g, followed closely by T3 (75 % *H. rosa-sinensis* + 25 % green tea), which showed 398.35 ± 2.09  mg GAE/100  g. The lowest TPC was recorded in T1 (25 % *H. rosa-sinensis* + 75 % green tea), with 271.98 ± 4.09  mg GAE/100  g. The observed results suggest a synergistic effect of green tea and *H. rosa-sinensis* extract at specific ratios, particularly in T3. Green tea is well-documented for its high phenolic content, especially catechins, which likely contributed to the elevated TPC in the control (T0). However, the substantial TPC in T3 indicates that *H. rosa-sinensis* flower is also rich in phenolic compounds and, when used at higher concentrations, can nearly match the antioxidant potency of green tea. These findings are consistent with Paraiso et al. [40] who found that freshly prepared hot infusions of *H. rosa-sinensis* had significantly higher TPC than cold or stored tea, likely due to better extraction efficiency of polyphenols at elevated temperatures. Another study conducted by Nguyen et al. [41] also reported that the dried calyces of *H. rosa-sinensis* contained high levels of phenolics (25.196  mg GAE/g), though the TPC was reduced in tea brewed at 90 °C (12.7  mg GAE/g), possibly due to thermal degradation or suboptimal extraction conditions.

Results of DPPH (%) inhibition of the *H. rosa-sinensis* flower extract based functional tea formulations with different treatment combination are presented in Fig. 4 **(b)**. The control green tea only, showed highest value 77.223 ± 1.25 % which may be due to the presence of high catechin content, while the lowest DPPH (%) inhibition was observed in T1 which is 53.85 ± 1.39 %. In the treatment groups highest DPPH (%) inhibition was observed in T3 of the functional tea 70.05 ± 1.48 %, this increases in the DPPH (%) inhibition maybe because of the synergistic effect between the *H. rosa-sinensis* flower extract and green tea. High DPPH (%) inhibition is because of the presence of flavonoids, anthocyanins and phenolic acids. Our findings of the DPPH (%) inhibition are coincided with the results of Paraiso et al. [40] who examined the antioxidant potential of *H. sabdariffa* calyces tea and reported that calyces hot tea can scavenge 218 mM TE/g of the DPPH radical, this value was the highest for the raw control that was used in the study for the hot tea infusion. Findings from the study of Nguyen et al. [41] showed similar results to our study for the DPPH (%) inhibition who found that the brewing the *H. sabdariffa* calyxes with hot water for the tea yielded 73.3 % of the DPPH (%) inhibition. Another study that was conducted by Widowati et al. [42] of the *H. sabdariffa* tea also coincided with our study where the DPPH (%) inhibition was found to be at 97 %.

The results of FRAP activity of *H. rosa-sinensis* flower extract based functional tea formulations with different combination are demonstrated in Fig. 4 **(c)**, showing significant (*p* > 0.05) difference in values. The green tea only demonstrated the highest FRAP activity 1759.0 ± 4.48 µgFe, followed by the T3 treatment whose FRAP activity value was observed as 1459.2 ± 5.94 µgFe/100 g. The lowest value for the FRAP activity was observed in T1 as (949.8 ± 5.48 µgFe/100 g). Highest reducing potential is possibly due to the presence of catechins and flavonoids which reduce the ferric ions [43]. While for the *H. rosa-sinensis* flower extract based functional tea formulations the contribution for the reducing power may be because of the organic acids and anthocyanins [44]. A study was conducted by Widowati et al. [42] evaluated the antioxidant activity of *H. sabdariffa* tea using the FRAP assay. The results indicated that at a concentration of 5 %, *H. sabdariffa* tea exhibited 17 % FRAP antioxidant activity.

ABTS antioxidant activity of whole *H. rosa sinensis* flower extract functional tea formulations with different treatment combination is displayed in Fig. 4 **(d)**, exhibited variations in values in various treatments. The highest value of ABTS was observed in the T3 treatment which is 71.79 ± 1.54 %, whilst the lowest value 47.69 ± 2.04 % was shown in negative control. T4 results were also at par with the T3 treatment combination of the functional tea. Our results are in close proximity with the findings of Paraiso et al. [40] who determined the ABTS (µM TE/g) of the *H. sabdariffa* calyces tea infusion at 85 (µM TE/g) for the raw fresh tea. ABTS activity of *H. sabdariffa* tea was evaluated by Widowati et al. [42] and the findings showed that *H. sabdariffa* tea exhibited 31 % in ABTS free radical scavenging assay.

### 3.5 Calorimetry values (L*, a*, b*) of *H. rosa-sinensis* flower extract based functional tea formulations

Color is a key sensory characteristic in evaluating tea infusions. In this study, color parameters were measured using the CIELAB system (L*, a*, b*). In this system, L* indicates lightness, with values ranging from 0 (black) to 100 (white); a* represents the red–green axis, where positive values indicate red and negative values indicate green; and b* represents the yellow–blue axis, where positive values indicate yellow and negative values indicate blue.

The results (Table 3) showed significant differences among treatments (*p* < 0.05). The control sample (T0, 100 % green tea) recorded the highest L* value, indicating it was the lightest in color. As the proportion of *H. rosa-sinensis* flower extract increased, L* values progressively decreased, producing darker infusions. For instance, T2 had an L* value of 48.15 ± 1.35, giving it a medium reddish-brown shade, while T4 had the lowest L* value, indicating the darkest infusion. This trend is consistent with the findings of Ramirez-Rodrigues et al. [45] who reported L* values between 35.26 and 54.18 for *H. rosa-sinensis* hot-water extract depending on extraction time. The a* values increased steadily with higher *H. rosa-sinensis* content, reflecting a stronger red hue due to anthocyanins. T0 showed the lowest red intensity (a* = 0.33 ± 0.05), while T1 had a higher a* value (9.42 ± 0.11), introducing a subtle red tint. In T3 and T4, the a* values were markedly higher, producing an intense red coloration characteristic of anthocyanin-rich infusions. This increase in redness aligns with previous study of Mejía et al. [14] who noted that anthocyanin-rich extracts impart vivid red tones in beverages. The b* values decreased as *H. rosa-sinensis* extract content increased, indicating a reduction in yellow tones. T0 exhibited the highest b* value, consistent with the yellowish color typical of green tea. T1 showed a slight decrease in b*, while T2 and higher treatments demonstrated further reductions, leading to a more balanced or red-dominant hue. The lowest b* values in T4 corresponded to the darkest, most reddish infusion with minimal yellow contribution. The color data collectively demonstrate that substituting green tea with *H. rosa-sinensis* flower extract decreases lightness, increases redness, and reduces yellowness. These changes are consistent with the known anthocyanin pigment profile of *H. rosa-sinensis*, which intensifies red tones while darkening the infusion and diminishing yellow shades. The combined sensory and instrumental results confirm that the proportion of *H. rosa-sinensis* strongly dictates the visual appeal and color intensity of functional tea blends.

**Table 3:** Colour analysis of *H. rosa-sinensis* flower extract based functional tea formulations prepared with different ratios.

**Treatment**	L*	a*	b*
T0	67.55 ± 1.96^a^	0.33 ± 0.05^e^	27.81 ± 2.69^a^
T1	57.81 ± 2.13^b^	9.42 ± 0.11^d^	22.00 ± 0.91^b^
T2	48.15 ± 1.35^c^	17.17 ± 0.17^c^	15.69 ± 0.28^c^
T3	31.32 ± 1.39^d^	26.28 ± 0.90^b^	11.20 ± 0.30^d^
T4	28.83 ± 0.39^d^	30.60 ± 0.59^a^	5.65 ± 0.18^e^

T0 (Control): 100 % green tea, T1: 25 % *H. rosa sinensis* flower extract + 75 % green tea, T2: 50 % *H. rosa sinensis* flower extract + 50 % green tea, T3: 75 % *H. rosa sinensis* flower extract + 25 % green tea, T4: 100 % *H. rosa sinensis* flower extract. Data are expressed as means ± SD. Values with different superscript letters in each column are significantly different (*P* < 0.05).

### 3.6 FTIR analysis of *H. rosa-sinensis* flower extract based functional tea formulations

**Fig. 5:**
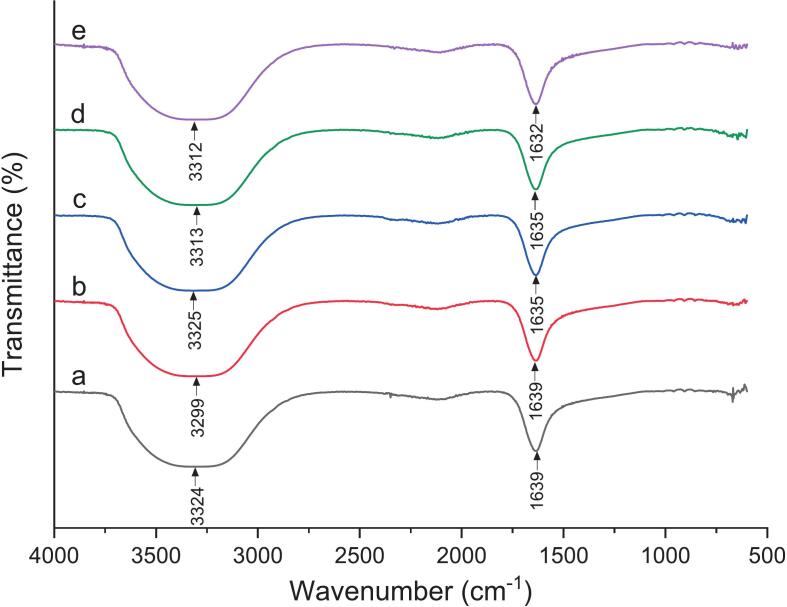
FTIR spectra of *H. rosa-sinensis* flower extract based functional tea formulations. (a) T0 (Control): 100 % green tea, (b) T1: 25 % *H. rosa sinensis* flower extract + 75 % green tea, (c) T2: 50 % *H. rosa sinensis* flower extract + 50 % green tea, (d) T3: 75 % *H. rosa sinensis* flower extract + 25 % green tea, (e) T4: 100 % *H. rosa sinensis* flower extract.

FTIR spectra (Fig. 5) revealed the characteristic vibrations associated with polyphenols, flavonoids, and related phytochemicals present in *H. rosa-sinensis* flower extract based functional teas. The spectrum of 100 % green tea (control) as shown in Fig. 5 **(a)** showed a prominent broad band at 3324 cm^−1^ corresponding to O–H stretching, confirming the abundance of hydroxyl groups typical of catechins and other tea polyphenols. A strong band near 1639 cm^−1^, attributed to C=C stretching of aromatic rings, reflected the presence of flavonoids, while peaks at 2850–2921 cm^−1^ indicated aliphatic C–H stretching. Incorporation of *H. rosa-sinensis* extract at 25 % concentration in tea produced broader O–H absorption (≈3299 cm^−1^) present in Fig. 5 **(b)**, reflecting enhanced hydrogen bonding due to phenolic hydroxyls such as quercetin and chlorogenic acid derivatives. Peaks at 1639 and 1014 cm^−1^ represented C=C and C–O stretching, respectively, confirming the coexistence of flavonoids and glycosidic linkages from anthocyanin conjugates. At 50 %, the O–H stretching peak shifted slightly to 3325 cm^−1^ with moderate intensity, indicating further hydrogen-bond formation (Fig. 5 **(c)**). A distinct band at 663 cm^−1^ suggested aromatic C–H bending, reinforcing the presence of phenolic aromatic structures. The 75 % extract based tea exhibited stronger spectral features, with an intense broad O–H band at 3313 cm^−1^ and C=C absorption at 1636 cm^−1^ (Fig. 5 **(d)**). The increased intensity of these peaks reflected higher concentrations of anthocyanins, phenolic acids, and glycosides (e.g., cyanidin-3-sambubioside). A pronounced C–O stretching band at 1011 cm^−1^ further supported the accumulation of glycosidic compounds contributing to antioxidant activity. The 100 % *H. rosa-sinensis* flower extract based functional tea showed the most intense O–H (3312 cm^−1^) and C=C (1632 cm^−1^) peaks, consistent with maximum phenolic and anthocyanin content. Minor C–H peaks (2840–2930 cm^−1^) indicated aliphatic structures, and the C–O band near 1011 cm^−1^ corresponded to esters and ethers derived from organic acids (Fig. 5 **(e)**). The progressive enhancement of O–H and C–O bands across formulations confirms the enrichment of hydroxylated and glycosylated phenolics, correlating with antioxidant potential. These results are consistent with previous *H. sabdariffa* FTIR studies [[45], [46], [47], [48]], confirming *H. rosa-sinensis* as a rich source of polyphenolic bioactives.

### 3.7 Sensory analysis of *H. rosa-sinensis* flower extract based functional tea formulations

**Fig. 6:**
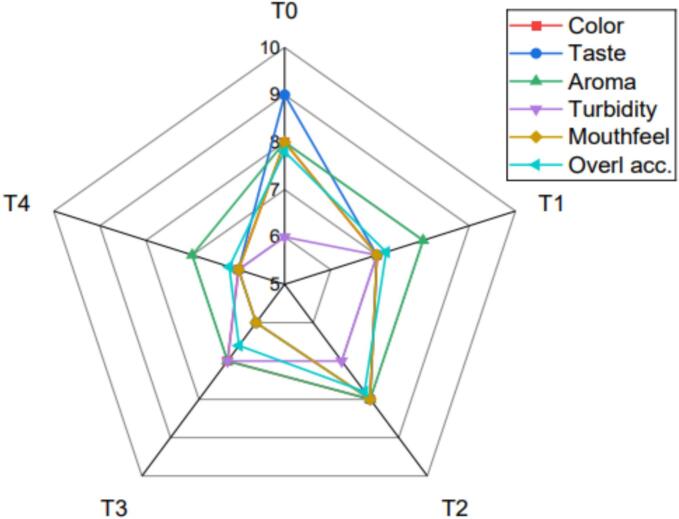
Sensory parameters of *H. rosa-sinensis* flower extract and green tea combination based functional tea formulations. T0 (Control): 100 % green tea, T1: 25 % *H. rosa-sinensis* flower extract + 75 % green tea, T2: 50 % *H. rosa-sinensis* flower extract + 50 % green tea, T3: 75 % *H. rosa-sinensis* flower extract + 25 % green tea, T4: 100 % *H. rosa-sinensis* flower extract.

The radar chart presented in Fig. 6 shows the sensory profiling of functional tea formulations of *H. rosa-sinensis* flower extract, which is blended with green tea at different proportions. The scores are described on chart on a scale of 0–9, as the higher scores indicate better sensory product attributes. The chart contains six parameters, such as taste, color, turbidity, mouthfeel, aroma and overall acceptability. As far as the color is concerned, T4 and T2 based functional tea got the highest score of 8.0 (Fig. 6). This may be due to the presence of anthocyanin pigments in *H. rosa-sinensis* flower, bestowing a bright color to the tea as compared to green tea which impart pale color [14]. These findings indicate that T4 significantly increased anthocyanin content which is evident with the high visual appeal and color intensity. However, the same high anthocyanin content for the taste parameter, T4 had the lowest score of 6.0 for taste, because of the excessive flower’s tartness, making it less favorable to the taste buds. This is because of the high organic acids like citric acid and polyphenolic content which contribute to increased sourness and lesser overall palatability [15,46]. However, the T2 scored highest 8.0, demonstrating an optimal balance between tartness and green tea acridness. Similar trend was observed with the aroma of functional tea formulations, the T2 got the highest score of 8.0. Furthermore, the characteristic aroma may be because of synergistic interaction between the floral *H. rosa-sinensis* scent and green tea vegetal aroma notes. It was also shown that the T0 or T4 did not produce desired smell, possible due to the single aromatic note from both teas.

Turbidity signifies the cloudiness or clarity in tea blends. The highest score was given to highest clarity or lowest turbidity treatment, which in current treatments was noticed in the T1 and T0. The higher value of turbidity in the functional teas with higher *H. rosa-sinensis* extract indicates high extraction rate of color pigments and total solids thereby leading to increased turbidity. Moreover, the T2, as well as T1, received the highest scores of 7.0, which indicates the smooth and pleasant texture regarding mouthfeel. Furthermore, T4 was slightly astringent and thicker, attenuating its palatability to some extent. Overall, the T2 scored highest (7.8) in terms of overall acceptance, highlighting a moderate combination of taste, color, aroma and mouthfeel as consumer inclination.

The sensory evaluation of various combinations reveals that T3 and T4 achieved highest points 8.0 of color tonality due to the presence of anthocyanins, which is red pigments in the hibiscus flowers. Moreover, it has been documented in numerous studies that the hot water usage in the preparation of functional tea or hot beverages results in higher extraction rate of anthocyanin, as hot water subsequently yields deeper and brighter color extracts [47]. Regarding the aroma and taste, the synergistic combination of both plants was more pronounced as T2 resulted in the highest flavor and aroma score because of the synergistic balance regarding the floral volatiles and tartness of *H. rosa-sinensis* and astringency of green tea besides the vegetal terpenes based elevated aroma. This was due to the single aromatic treatment in the T4 resulted in a lower acceptance score of 6.2, indicating the importance of blending [7]. Most preferred formulation in our study was with the balanced *H. rosa-sinensis* extract proportion with moderate acidity and retention of enough anthocyanin content for desirable color, consumer acceptance and antioxidant capability. This is evident by the studies of blends of *H. rosa-sinensis* and green tea, where a moderate ratio of ingredients enhanced the consumer acceptance, with balanced sourness while also maintaining functional quality [48]. Some studies on the blends of green tea and *H. sabdariffa* (3:7 v/v) have reported synergistic enhancement with respect to the phenolic stability, antioxidant capacity and sensory acceptance. Similar results were found in another study which demonstrated synergistic effect of *H. sabdariffa* with decaffeinated green tea in maintaining sensory and antioxidants activity in the formulation of functional tea [49,50]. Another study was conducted to measure the hibiscus astringency, flavor intensity, and odor which attributed that such parameters are relatively higher in hibiscus infusions as compared to the syrup. Moreover, this effect also increased with the increase in the hibiscus percentage. It has been observed that the intensity of sweet taste was comparatively low due to the unique formulations. Furthermore, the increase of *H. rosa-sinensis* extract in functional tea resulted in higher astringency factor, clarity, and higher turbidity, as T3 and T4 had higher astringency and turbidity characteristics, which is attributed to high proportion of anthocyanins, dissolved solids (i.e. polysaccharides).

## 4 Conclusion

Despite the high nutritional and bioactive potential of certain edible flowers, many remain underutilized in functional food and beverage development. *H. rosa-sinensis* is one such flower, rich in minerals, polyphenols, and antioxidants, yet its potential for functional formulations has not been fully explored. This study aimed to evaluate the nutritional and bioactive profile of *H. rosa-sinensis* flower and assess their suitability for developing functional tea. The research was conducted in two phases. In the first phase, the flowers were analyzed for proximate composition, mineral profile, TPC, and antioxidant activity using extracts obtained via UAE. In the second phase, functional tea formulations were formulated with varying ratios of *H. rosa-sinensis* flower extract and green tea. These teas were evaluated for TPC, antioxidant potential (via DPPH, ABTS, and FRAP assays), color attributes (L*, a*, b*), functional group composition using FTIR, and sensory properties assessed by an expert panel. The mineral profile indicated that *H. rosa-sinensis* flower is rich in calcium, and potassium. The flower also exhibited high TPC and antioxidant capacity. Among tea formulations, the T0 which is only green tea had the highest TPC and FRAP activity, whereas the formulation with 75 % *H. rosa-sinensis* extract (T3) showed the strongest DPPH and ABTS radical scavenging activity. Colorimetric analysis revealed that the 50 % *H. rosa-sinensis* formulation (T2) offered the most balanced color profile, which corresponded with the highest sensory acceptance due to its moderate redness, slightly acidic taste, and vibrant aroma. In contrast, the 100 % *H. rosa-sinensis* tea was considered too sour and intensely red, likely due to higher anthocyanin content and astringency. FTIR spectra across all formulations showed common functional groups such as O–H stretching (hydroxyl groups from polyphenols), C=C (flavonoids), and C–H (organic backbone), with higher *H. rosa-sinensis* extract proportions showing stronger absorbance in antioxidant-associated bands. These results highlight *H. rosa-sinensis* as a valuable ingredient for functional tea development, offering nutritional, antioxidant, and sensory benefits. The findings support its potential as a nutraceutical beverage ingredient, contributing to health-oriented product innovation. The present study was limited to in vitro antioxidant assays and sensory evaluation under controlled laboratory conditions. To fully validate the health benefits and functional properties of *H. rosa-sinensis* based functional tea, future studies should include in vivo trials. Additionally, industrial-scale processing trials, shelf-life assessments, and market analyses are recommended to evaluate commercial feasibility, consumer acceptance, and product stability. Such investigations will facilitate the translation of these findings into large-scale production and support the development of a novel, health-promoting functional beverage with clean-label appeal.

Compliance with ethical standards

Research which involves human or animals

Sensory analysis of the functional tea involving the human participants was conducted in compliance with the ethical guidelines. Ethical approval was acquired from Research Ethics Panel of the Institutional Review Board with Approval No. 110/25, dated 27 March 2025. Furthermore, consent was acquired from all participants before the evaluation to ensure cohesion to ethical standards.

Declaration of generative AI in scientific writing

During the preparation of this work, the authors used ChatGPT to improve readability and language. After using this tool, the authors reviewed and edited the content as needed and they accept full responsibility for the content of the publication.

## CRediT authorship contribution statement

**Hassan Raza:** Writing – original draft, Visualization, Methodology, Data curation, Conceptualization. **Muhammad Tauseef Sultan:** Writing – review & editing, Supervision, Formal analysis. **Samran Khalid:** Writing – review & editing, Writing – original draft, Formal analysis, Data curation, Conceptualization. **Muhammad Usman Khalid:** Writing – review & editing. **Kashmala Chaudhary:** Writing – original draft, Visualization, Formal analysis, Data curation. **Shehnshah Zafar:** Writing – review & editing. **Ahmad Mujtaba Noman:** Writing – review & editing. **Ahmad Raza:** Writing – review & editing. **Helen Onyeaka:** Writing – review & editing, Funding acquisition, Formal analysis.

## Declaration of competing interest

The authors declare that they have no known competing financial interests or personal relationships that could have appeared to influence the work reported in this paper.
